# The genetics of adipose tissue metabolism

**DOI:** 10.1098/rsos.231478

**Published:** 2024-02-07

**Authors:** Rebecca Dumbell, Roger D. Cox

**Affiliations:** ^1^ Dept of Biosciences, School of Science and Technology, Nottingham Trent University, Clifton Lane, Nottingham NG11 8NS, UK; ^2^ MRC Harwell Institute, Mammalian Genetics Unit, Harwell Campus Oxfordshire, Harwell OX11 0RD, UK

**Keywords:** adipose, genetics, waist–hip ratio, organoids

## 1. Introduction

Most recent figures estimate that 39% of the world's population is overweight, with 11% of men and 15% of women being defined as having obesity [1], and in 2014 this was estimated to have a global financial cost estimated to exceed $2.0 trillion (2.9% global GDP) [2]. Obesity is a leading contributor to preventable death, with comorbidities including metabolic disease such as type 2 diabetes, cardiovascular disease, some cancers, and poorer outcomes from COVID-19 infections associated with overweight and obesity [3–5]. With advancing technology and methodologies, we are now better able than ever before to understand the underlying genetics that contribute to adipose physiology and its role in obesity and links to metabolic disease. In this perspective we will discuss why it is important to understand genetic regulation of adipose physiology, what we currently know, and where this is likely to lead in future developments.

## 2. Adipose tissue genetics

Adipose tissue has a diverse range of functions from biomechanical to multiple important roles in physiology. Biomechanical roles include for example, shock absorption in joints, feet and palms as well as roles in forming facial structures [6]. Physiologically, adipose tissue can be categorized into different general types. White adipose tissue (WAT) is the ‘classic’ energy storage tissue, containing a single large lipid droplet per cell, storing energy as triglycerides which are liberated as free fatty acids (FFAs) when required. Brown adipose tissue (BAT) is found in infants and in discrete depots in some adults, contains smaller cells densely packed with mitochondria and can generate heat through non-shivering thermogenesis fuelled by uncoupling the electron transport chain. Beige or brite adipocytes have an intermediate phenotype and are found in mainly subcutaneous WAT depots and can function much like a brown adipocyte. This is a broad categorization, and the role, function and lineage of these cells are an active area of investigation, which is discussed in detail elsewhere [6,7].

The metabolic importance of WAT is shown by patients with lipodystrophies, a heterogeneous disorder in which there is a partial or generalized lack of WAT and adipose tissue dysfunction [8,9]. This results in severe insulin resistance, dyslipidaemia and non-alcoholic fatty liver disease (NAFLD). Lipodystrophy may be acquired but more than 20 genes are linked to monogenic lipodystrophy syndromes and affect adipose tissue directly [10]. These include, for example, key regulators of adipose cell differentiation and tissue function such as the master regulator *PPARg* that is mutated in familial partial lipodystrophy (FPLD3) [11,12]. There is also evidence that some cases of lipodystrophy, familial partial lipodystrophy 1 (FPLD1), may have a polygenic basis [13,14]. Lipodystrophies link the effect of genetic changes on intrinsic adipose tissue function, to changes in fat distribution and consequently metabolic syndrome.

Body fat distribution, measured indirectly through waist circumference–hip circumference ratio (waist–hip ratio, WHR) is a more reliable indicator of disease risk than Body Mass Index (BMI; body mass divided by the square of height, kg m^2^) alone [15]. A recent meta-analysis in the *British Medical Journal* indicated that central obesity, measured by WHR and other techniques, was associated with a higher all-cause mortality risk [16]. Further, the pooled relative risk per standard deviation of WHR for type 2 diabetes in a meta-analysis of 32 publications [17] was 1.88 (95% CI: 1.61, 2.19). Additionally, the hazard ratio (adjusted) for cardiovascular disease incidence in the EPIC-Norfolk prospective study for the top quartile of WHR in women and men was 1.46 (1.27 to 1.69) and 1.26 (1.10 to 1.43) respectively [18].

Simple measures of WHR are highly correlated with BMI, requiring adjustment, and the relationship between WHR and specific fat depot differences as well as variation in muscle and bone is not identified. However, depot volumes can be determined directly using dual-energy X-ray absorptiometry (DXA) or magnetic-resonance imaging (MRI) [14,19]. These measures have been used to predict disease risk either using direct or indirect measures or by genetic association (see below) and determining genetic risk scores. In a study using UK Biobank (UKBB), DXA data from 4198 individuals was used to train models using anthropometric and bioelectrical impedance data to *predict* visceral adipose tissue (VAT) in 400 000 UKBB individuals [19]. When adjusted for BMI, predicted higher VAT showed higher sexually dimorphic risk for type 2 diabetes (women odds ratio (OR) 3.69 (3.36 to 4.05) and men OR 1.78 (1.67 to 1.89)), heart attack/angina (women OR 1.60 (1.421 to 1.81) and men 1.26 (1.18 to 1.34)) and hypertension (women 1.74 (1.66 to 1.83) and men 1.29 (1.25 to 1.34)) [19].

Body fat distribution has a genetic component, and genome-wide association studies (GWAS) have identified more than 340 loci associated with WHR and WHR adjusted for BMI, almost exclusively in regulatory and non-coding regions of the genome [20–23]. Unlike with BMI, these WHR loci do not tend to be associated with genes known to regulate energy balance centrally and are more likely to have peripheral action in adipose tissue, representing differences in adipose depot function and fat distribution [21]. Recent direct analysis of depot volumes using MRI and genetic association has been carried out on 38 965 UKBB individuals. This study determined genetic associations for multiple traits including visceral (VAT), abdominal subcutaneous (ASAT) and gluteofemoral (GFAT) adipose tissue and adjusted (adj) for age, BMI and height to identify 250 independent common variants (39 of them new) [14]. Importantly, this precision depot-specific approach shows distinct depot patterns of associated genes further facilitating efforts to identify new variant to function biology. Finally, using these MRI-derived genetic associations allows depot-specific disease risk to be evaluated in unimaged individuals using polygenic risk scores (PRS). For example, amongst 447 486 individuals in UKBB that are within the bottom 5% PRS for gluteofemoral fat (GFATadj) there was an increased risk for type 2 diabetes (OR 1.29 (1.23 to 1.35)), coronary artery disease (OR 1.09 (1.04 to 1.14)) and hypertension (OR 1.13 (1.09 to 1.16)). This is consistent with the view that inadequate expansion of the GFAT depot results in metabolically unhealthy fat distribution [14].

These studies have shown that fat distribution, independent of BMI, is highly heritable, depot-specific and clearly associated with disease risk. Support for a causal relationship between fat distribution and disease risk has been shown using Mendelian randomization studies in the UK Biobank [24,25].

It is thought that the effect of changes in fat distribution on metabolic disease risk is due to differing physiological function of subcutaneous and visceral WAT depots, and that people with higher WHR or central obesity having a higher ratio of visceral : subcutaneous WAT. Since it is the visceral WAT that tends to be blamed for deleterious effects of obesity, high WHR can also be considered a lower ratio of ‘safer’ subcutaneous lipid stores, which are found in the hips and limbs. Thus, lower body fat is more protective relative to visceral fat [26]. However, inadequate expansion of the subcutaneous depots for lipid storage, leading to lipid deposition in visceral depots and ectopically may actually be the critical factor contributing to the disease risk associated with visceral depots. This is shown by Mendelian randomization studies using genetic instruments for adipose depots from Agrawal *et al*. [14], demonstrating that GFATadj is strongly associated with cardiometabolic traits and disease, higher than for VATadj and ASATadj [25].

The selective pressures leading to the evolution of diversified adipose tissue depot function and the advantages conferred on the whole organism are interesting questions. An example of selection during the evolution of a WHRadjBMI locus is provided by the *WARS2/TBX15* WHRadjBMI locus [21] with evidence of introgression of Archaic Denosovian DNA in Greenland Innuit and Native American populations [27]. This correlates with *WARS2* and *TBX15* expression changes, potentially mediated through differences in methylation patterns, that may be relevant to selection acting on the pleiotropic effects of this locus (including in adipose tissues) during the temporally extended migration of ancient human populations through the Northern Hemisphere to populate the Americas [27]. The *TBX15* gene is linked to adipose differentiation and browning [28–30] and *WARS2* to mitochondrial function [31–33], therefore altered expression could contribute to biological adaption to the low temperatures and the specific diet of the Arctic.

## 3. Variants to function

The challenge, to exploit this tremendous genetic resource of information, is to link variants from GWAS with the regulation and function of specific genes and with specific mechanisms. There are multiple approaches to achieving these aims, combining human genetic information, imaging and physiological assays in individuals of known genotype, manipulation of cells and cell lines, gene editing of inducible pluripotent stem cells, analysing cells and tissues from individuals of known genotype and use of animal models. Divergence (from human) of regulatory pathways in species such as the mouse complicates modelling of human complex disease mechanisms, where differences in gene regulation may be key [34].

The complexities of these approaches are exemplified by the *FTO* BMI locus discovered in 2007, which probably involves the regulation of multiple genes in developmental, tissue and cell-specific contexts to exert its effect on BMI [35–39]. To increase the rate of translation of these loci, new approaches are probably required to be integrated within the existing pipelines.

## 4. Modelling adipose tissue

With the success of modelling tissues using organoid culture systems, particularly the developing brain [40,41], there are opportunities for similar approaches to adipose tissue. Adipose tissue has a distinctive structure depending on the depot and is composed of multiple cell types. There is a complex interaction in adipose tissue between lipid storing cells, cells of the immune system, the sympathetic nervous system and vasculature. These interactions in adipose tissue are central to disease and ageing processes, for example inflammatory processes leading to insulin resistance [42]. However, conventional two-dimensional adipocyte cultures of cell lines or primary cells isolated from adipose tissue exhibit multi-locular lipid droplets and show limited physiological behaviours; and in the case of mixed primary cultures do not support survival of different cell types and their interactions. Two-dimensional cultures have the advantage of being simple and scalable, easy to manipulate for example to knock out gene expression, and to make functional correlations using lipid content and gene expression assays.

Three-dimensional adipose cell cultures in spheroids and other types of support matrix offer the potential to develop an organoid that better represents the intrinsic three-dimensional structure and function of adipose tissue, with vascularization, immune cells and progenitors. Several systems have been described that offer the opportunity for investigating more complex physiology *in vitro,* although these systems do not yet fully recapitulate adipose tissue and indeed are comprised of single cell types or mixtures of cells from primary tissue isolation [43–46]. These three-dimensional models show improved differentiation with larger lipid droplets and may offer high throughput applications with better modelling of tissue. However, considerable further development is still required to address the challenges of developing a true adipose tissue organoid. Further, the ongoing challenge for adipose organoids is to develop a reproducible and properly functioning tissue homologue by introducing the necessary progenitor cells to an appropriate structural environment that allows for self-organization, differentiation and development under culture conditions that retain cellular diversity, differentiation and the survival of a pool of cells capable of further proliferation and differentiation.

To be useful in exploring adipose physiology, these new organoid systems will require detailed characterization to determine which cell types are present, how they change over time and treatment and how they compare in function with cells within *in vivo* tissue. This could be partly addressed using single cell RNA sequencing approaches, and by the application of high content imaging such as the recently described LipocyteProfiler applied to organelle labelled adipocyte cells [47]. Further, crucial to their application, adipose organoids need to exhibit tissue-like lipogenic and lipolytic properties in response to stimuli, hyperplastic and hypertrophic responses to storage demand, tissue inflammatory states, visceral and subcutaneous depot differences (as well as brown and beige) and regulated adipokine secretion. In addition to the assays outlined above, these may be determined for example using imaging of lipid droplets to measure lipid content and collect morphometric data, response to fatty acid feeding, fluorescent glucose uptake assays in the presence or absence of insulin, FFA and glycerol secretion, adipokine and inflammatory cytokine secretion assays, imaging of inflammatory cells and oxygen consumption with or without stimuli. These metabolic readouts can be correlated with similar *in vivo* readouts. Ultimately, standardization of adipose organoids is also required so that they can be used robustly and reproducibly applied in different laboratories.

A functional adipose organoid has many applications. To investigate the genetics of, and candidate genes for, conditions that affect adipose tissue and that determine fat distribution and adipose depot function, cells could be genetically manipulated using CRISPR in induced pluripotent stem cells (iPSCs), primary cells isolated from individuals of selected genotype or from genetically modified organisms. Importantly, Laber *et al.* have shown proof of principle for genetic variation in primary cultured adipocyte precursors to produce genotype specific cellular and morphological profiles using LipocyteProfiler [47]. These approaches may be particularly useful in the discovery of functional GWAS variants and their effects on gene expression and cellular physiology.

Whole organoids might also be targeted for genetic alteration using libraries of viral gene silencing systems for example, for a genome-wide screen of gene function. The effect of genetic changes would then be assayed in a battery of parallel functional assays to provide a broad functional assessment and target discovery. Application of high throughput robotic organoid production, automated handling in 96 and higher well formats and assaying would facilitate compound screening and evaluation of toxicity [44]. Further, as inflammatory processes and senescence are an important factor in metabolic health and ageing, including in adipose tissue, an organoid model that recapitulates these features could be useful in screening therapeutics [48,49].

## 5. Perspective

It is apparent from GWAS for WHRadjBMI and depot specific volume, that adipose tissue and its environmental interactions are under some significant degree of genetic regulation, and given the association of fat distribution with metabolic disease, there is potential for new mechanisms and therapeutic interventions if these associations can be translated into mechanisms. Given the challenges in translating genetic associations to their mechanistic function, a combination of approaches that overcome traditional *in vitro* and *in vivo* genetic strategies are required to decipher what drives these associations and to design new interventions for the prevention of disease.

## Data Availability

This work did not require ethical approval from a human subject or animal welfare committee.

## Data accessibility

This article has no additional data.
